# Phylogenetic and morphological discord indicates introgressive hybridisation in two genera of Australian millipedes (Diplopoda, Polydesmida, Paradoxosomatidae)

**DOI:** 10.3897/zookeys.809.30087

**Published:** 2018-12-19

**Authors:** Peter Decker

**Affiliations:** 1 Senckenberg Museum of Natural History Görlitz, Am Museum 1, 02826 Görlitz, Germany Senckenberg Museum of Natural History Görlitz Görlitz Germany

**Keywords:** Aberrant morphology, Arthropoda, COI, hybridisation, introgression, Myriapoda, 16S, 28S

## Abstract

Discord between molecular and morphological datasets was observed in two pairs of species of Australian millipedes in the family Paradoxosomatidae using morphological and molecular phylogenetic analysis (mitochondrial COI rDNA and 16 rRNA, and nuclear 28S rRNA). Close to the presumed distributional boundary between *Pogonosternumnigrovirgatum* (Carl, 1912) and *Pogonosternumjeekeli* Decker, 2017, near Dargo in Central Gippsland, Victoria, *Pogonosternum* specimens were collected which are phylogenetically closer to *P.jeekeli* in COI and 16S sequences, but are morphologically closer to *P.nigrovirgatum.* At Mount Osmond, Adelaide, South Australia, eight morphologically typical *Somethuscastaneus* (Attems, 1944) specimens were collected are phylogenetically closer to *S.castaneus* in 28S genealogy, but three of the eight are closer to *S.lancearius* Jeekel, 2002 in COI genealogy. These two cases are discussed in terms of hybridisation, past introgressive hybridisation events and aberrant morphology.

## Introduction

While many cases of hybridisation in plants, fungi and animals are well known (Alix et al. 2017, Crossman et al. 2016, Ottenburghs et al. 2016, Stukenbrock 2016, Yazicioglu et al. 2016), examples in millipedes are scarce. Pedroli-Christen and Scholl (1990) studied a hybrid zone between the two chordeumatidans *Rhymogonacervina* (Verhoeff, 1910) and *Rhymogonasilvatica* (Rothenbühler, 1899) in the Swiss Alps. However, later revisions (Pedroli-Christen and Scholl 1996; Scholl and Pedroli-Christen 1996) placed both nominal species within *R.montivaga* (Verhoeff, 1894), either as a synonym (*R.silvatica*) or as a subspecies (*R.cervina*).

In Illinois, United States, the monotypic polydesmidan *Illiniurusbeattyi* Shear, 1968 was later described as “transitional” in gonopod structure between *Euryurusleachii* Gray, 1832 and *Auturusevides* Bollman, 1887, both of which occur near the *I.beattyi* type locality (Jorgensen 2009). Later searches of the area for *I.beattyi* were unsuccessful, and Jorgensen (2009) was unable to determine whether the one known male of *I.beattyi* was a hybrid of *E.leachii* and *A.evides*, or simply an aberrant individual of one of these two euryurid species.

The introduction of genes from the gene pool of one species into that of another during hybridization, especially near species boundaries, is called introgressive hybridisation and can affect nuclear or mitochondrial DNA (Harper and Hart 2007, Harrison and Larson 2014, Toews and Brelsford 2012). Introgressive hybridisation has not yet been reported in the class Diplopoda.

Two cases of disagreement between relationships inferred from morphological similarity and molecular phylogenetics were observed in recent taxonomic studies of the Australian paradoxosomatid genera *Pogonosternum* Jeekel, 1965 (Decker 2016a, Decker et al. 2017) and *Somethus* Chamberlin, 1920 (Decker 2016b). These two cases are described here in detail and discussed with regard to hybridisation, past introgressive hybridisation events and aberrant morphology.

## Materials and methods

### Specimen collection and preservation

Pogonosternumcf.nigrovirgatum (Carl, 1912) “Dargo”: 7 males, 1 female, 1 juvenile (NMV K-12202, K-13866, K-13867, K-13474, K-13868, SMNG VNR018276, VNR018277) were collected by hand in forest on Dargo Road, SSW of Dargo, central Gippsland, Victoria, 37.595S, 147.193E by P. Decker, K. Voigtländer and R. Mesibov on 14 August 2014.

*Somethuscastaneus* (Attems, 1944): specimens were collected by hand at two localities in Mount Osmond Reserve, Adelaide, South Australia by P. Decker and K. Voigtländer : 1 male (SMNG VNR016973) and 3 females (SAM OM2149, SMNG VNR016975 and VNR016976) on a southwestern slope (34.969S, 138.654E, 27 August 2014, site number S110), and 5 males (SAM OM2138, SAM registration in progress, SMNG VNR018275) and 1 female (SMNG VNR018274) on a northern slope (34.962S, 138.659E, 23 August 2014, site number S90).

Specimens were killed and stored in 95% ethanol, with a change of ethanol after 1–2 months. One male of *Somethuscastaneus* from Mt. Osmond (SAM OM2138) was found dead in the field. DNA was not obtained from this specimen. The material is deposited in the Museums Victoria, Melbourne, Victoria, Australia (**NMV**), the South Australian Museum, Adelaide, Australia (**SAM**) and the Senckenberg Museum of Natural History Görlitz, Görlitz, Germany (**SMNG**).

### Illustrations

Preserved specimens were imaged with a Leica Z6 Apo stereo microscope and Leica DFC420 camera. Focus-stacked images were assembled from 25–40 source images using the software package Leica Application Suite 4.5. All images were later edited using Adobe Photoshop CS4 and assembled into plates. The distribution maps were created with ArcMap 10.

### Molecular analysis

DNA was extracted from 2–4 legs from each of four male Pogonosternumcf.nigrovirgatum “Dargo” and nine *Somethuscastaneus* specimens from Mt Osmond (Table 1). Total genomic DNA was extracted using a Qiagen DNAeasy Blood & Tissue kit following the standard protocol with an incubation of tissue for 48h. Glom primer cocktail pairs (Decker 2016a, 2016b, Macek et al. 2014) were used to sequence a 618 bp fragment of the mitochondrial cytochrome *c* oxidase subunit I (COI) gene. Primer pairs 28S D1a (Fw) and 28S D3b (Rv) (Dell’Ampio et al. 2009) were used to amplify 1225 bp of the D2 fragment and adjacent areas of D1 and D3 on the nuclear 28S ribosomal RNA gene. Primer pairs 16Sar (Fw) (5’-CGCCTGTTTAACAAAAACAT-3’) and 16Sbr (Rv) (5’-CCGGTCTGAACTCAGATCACGT-3’) (Simon et al. 1994) were used to sequence a 566 bp fragment of the large-subunit ribosomal RNA (16S) gene. For PCR protocol and all primer sequences (COI, 16S, 28S) see Decker (2016a, 2016b). Fragments were sequenced in both directions by the BiK-F Laboratory Centre, Frankfurt, Germany. All obtained sequences were checked with BLAST in GenBank and no contamination was apparent. The sequences were aligned by hand in ClustalX ver. 1.83 (Chenna et al. 2003) and uploaded to GenBank (Table 1). In addition, 41 previously published (Decker 2016a for more information) sequences for five *Pogonosternum* species *P.nigrovirgatum*, *Pogonosternumadrianae* Jeekel, 1982, *Pogonosternumlaetificum* Jeekel, 1982, *Pogonosternumjeekeli* Decker, 2017, and *Pogonosternummontanum* Decker, 2017 (COI: Genbank accession numbers KU745235–KU745274, KT948680; 16S: KU745194–KU745234) were used for the phylogenetic analysis of the *Pogonosternum*cf.nigrovirgatum “Dargo” sequences. For the molecular analysis of *Somethuscastaneus* “Mt Osmond” sequences the following published (Decker 2016b for more information) sequences were included: 16 COI sequences for the five *Somethus* species *S.castaneum*, *Somethusinflatus* (Jeekel, 2002), *Somethuslancearius* Jeekel, 2002, *Somethusscopiferus* Jeekel, 2002, and *Somethusgrossi* Jeekel, 1985 (GenBank accession numbers KT948655–KT948656, KT948658, KT948662–KT948670, KT948672–KT948676) and 15 28S sequences of the four species *S.castaneum*, *S.inflatus*, *S.lancearius*, and *S.grossi* (GenBank accession numbers KT964457–58, KT964457). *Archicladosomamagnum* Jeekel, 1984 (KT948681) was used as outgroup. Primary homologisation problems in the 16S rRNA sequences of the *Pogonosternum* dataset arose because of the highly variable expansion loops. As a result, selected alignment positions (272–297) were excluded from the 16S rRNA dataset. COI and 16S sequences were combined in *Pogonosternum* as a single dataset and incongruence assessed between them with the incongruence length difference (ILD) test (Farris et al. 1994) implemented as the partition homogeneity test in PAUP* version 4.0b10 using a full heuristic search, 10 random taxon addition replicates, tree-bisection-reconnection (TBR) branch swapping, and with MaxTrees set to 100 (Swofford 2002). The best-fit model of nucleotide substitution for the individual COI and 16S dataset was determined by MrModelTest 2 (Nylander 2004).

**Table 1.** Site numbers, localities, GenBank accession numbers and repository accession numbers for all specimens analysed. NMV = Museum Victoria, Melbourne, Victoria, Australia; SAM = South Australian Museum, Adelaide, Australia; SMNG = Senckenberg Museum of Natural History Görlitz, Görlitz, Germany; SA = South Australia; VIC = Victoria.

Species	Site No.	Locality	Sex	GenBank Acc. No. COI	GenBank Acc. No. 16S	GenBank Acc. No. 28S	Voucher
* Somethus castaneus *	S110-1	SA, Mt. Osmond, SW slope	male	KT948668		KT964470	SMNG VNR016973
	S110-2	SA, Mt. Osmond, SW slope	female	MK170142		MK142784	SAM OM2149
	S110-3	SA, Mt. Osmond, SW slope	female	MK170143		MK142785	SMNG VNR016975
	S110-4	SA, Mt. Osmond, SW slope	female	MK170144		MK142786	SMNG VNR016976
	S90-1	SA, Mt. Osmond, N slope	male	MK170145		MK142787	SAM
	S90-2	SA, Mt. Osmond, N slope	male	MK170146		MK142788	SAM
	S90-3	SA, Mt. Osmond, N slope	male	MK170147		MK142789	SAM
	S90-4	SA, Mt. Osmond, N slope	female	MK170148		MK142790	SMNG VNR018274
	S90-5	SA, Mt. Osmond, N slope	male	MK170149		MK142791	SMNG VNR018275
* Pogonosternum nigrovirgatum *	Dargo-1	VIC, SSW of Dargo	male	MK170150	MK170154		NMV K-12202
	Dargo-2	VIC, SSW of Dargo	male	MK170151	MK170155		NMV K-13866
	Dargo-3	VIC, SSW of Dargo	male	MK170152	MK170156		SMNG VNR018276
	Dargo-4	VIC, SSW of Dargo	male	MK170153	MK170157		SMNG VNR018277

Phylogenetic hypothesis was inferred for COI+16S, COI and 28S by using the maximum likelihood method conducted in MEGA6 (Tamura et al. 2011). The phylogenetic trees with the highest log likelihood (COI+16S: -5141; COI: -2565; 28S: -2328) are shown (Figs 3, 5). Initial trees for the heuristic search were obtained by applying the neighbour-joining method to a matrix of pairwise distances estimated using the Maximum Composite Likelihood (MCL) approach (Tamura et al. 2004). A discrete Gamma distribution was used to model evolutionary rate differences among sites (five categories (+G, parameter = COI+16S: 0.6793; COI: 0.1017; 28S: 0.0500)). The bootstrap consensus tree inferred from 1000 replicates (Felsenstein 1985) is here used as the best estimate of the phylogeny of each of the analysed taxa (Figs 3, 5). Mean uncorrected pairwise distances between terminals (transformed into percentages) were determined using MEGA6 (Tamura et al. 2011).

## Results

### Molecular analysis

The final alignments consisted of 618 bp of COI mtDNA and 1206 bp of 28S rRNA in *Somethus*, and 1158 bp for COI+16S in *Pogonosternum*. Individual alignments are available upon request from the author. The best-fit model of nucleotide substitution selected using MrModelTest 2 was the General Time Reversible model with gamma distribution and proportion of invariant sites (Nei and Kumar 2000) for the individual COI and 16S dataset. The trees constructed from individual genes did not show significant conflicts in topology (nodes different among trees with support > 70% in ML) and no significant incongruence among the three genes was revealed by the ILD test (*P* > 0.81 in all of the pairwise comparisons), and the sequences were concatenated into a dataset comprised 1158 characters for phylogenetic analysis in *Pogonosternum*.

### Morphology and sequence analysis of Pogonosternumcf.nigrovirgatum “Dargo”

**Figure 1.**
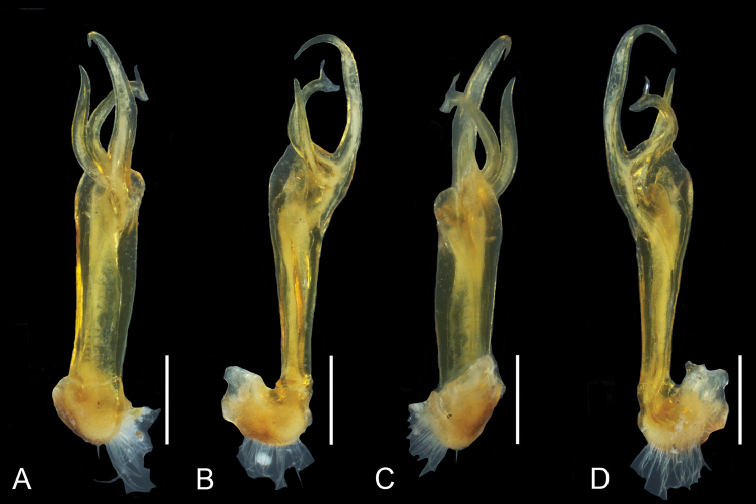
Pogonosternumcf.nigrovirgatum “Dargo”, male, right gonopod (NMV K-12202). **A** Posterior view **B** Lateral view **C** Anterior view **D** Mesal view. Abbreviations: *fp1* = femoral process 1; *fp2* = femoral process 2; *lp* = lateral process; *prof* = prolongation of femorite; *S* = solenomere; *F* = femorite. Scale bar: 0.5 mm.

**Figure 2.**
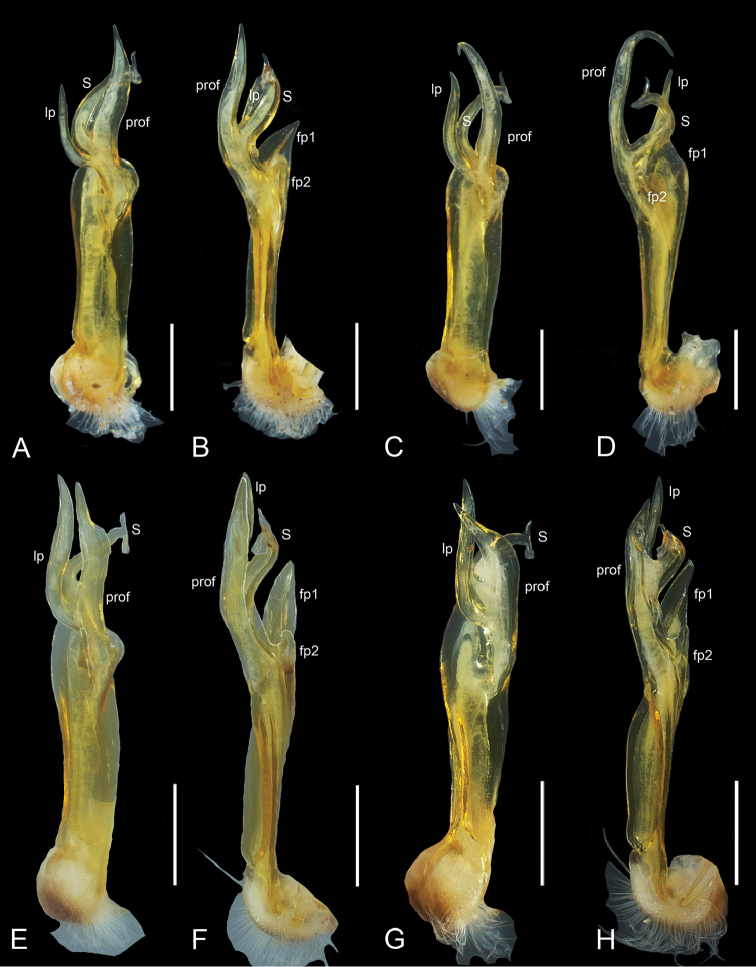
*Pogonosternumnigrovirgatum* (Carl, 1902), ♂, right gonopod. **A, B** AMS KS96017 Ferntree Gully **C, D**NMV K-10248 from Sandringham and Brighton. *Pogonosternumjeekeli* Decker, sp. n., ♂, right gonopod **E, F**NMV K-10252 from Dyer Creek **G, H**NMV K-10250 from Bemm River. **A, C** Posterior view **B, D** Mesal view. Scale bar: 0.5 mm.

**Figure 3.**
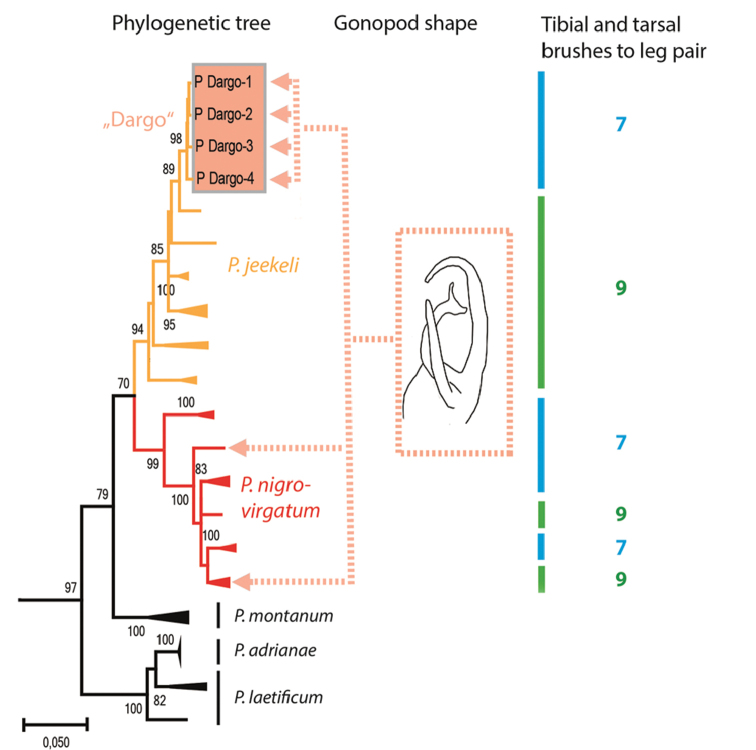
Bootstrap consensus tree for the combined mt COI+16S dataset in *Pogonosternum*; maximum likelihood, 1000 bootstrap replicates. Coloured dotted lines indicate specimens with similar gonopod morphology. Coloured bars indicate last male leg pairs with tibial and tarsal brushes (leg pair 7 = blue, leg pair 9 = green).

**Figure 4.**
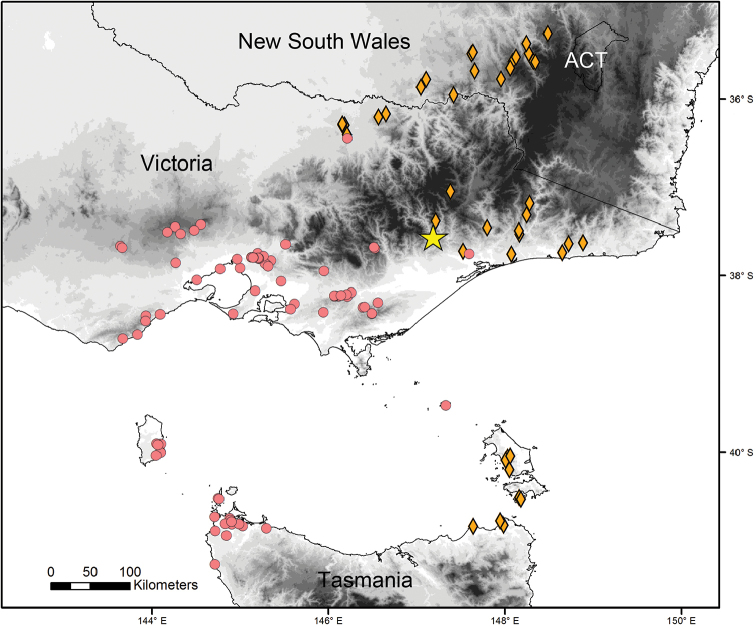
Distribution map of *Pogonosternumnigrovirgatum* (red circles), *P.jeekeli* (orange diamonds) and P.cf.nigrovirgatum “Dargo“ (yellow star) in southeastern Australia. Abbreviation: ACT = Australian Capital Territory.

*Pogonosternumnigrovirgatum* and *P.jeekeli* are very similar in somatic morphology, and the “Dargo” form agrees with both species in size, colouration, spiracle morphology and form of the leg pair 2 coxa in females. The “Dargo” form (Fig. 1) agrees with typical *P.nigrovirgatum* (Fig. 2) in having an elongated gonopod femorite and differs from *P.jeekeli* in gonopods (Fig. 3) and male tarsal and tibial brushes only on leg pairs 1–7. See also Decker et al. (2017) for a detailed (re)description of *P.nigrovirgatum* and *P.jeekeli* and the considerable gonopod variability in these species as well as Decker 2016a for phylogeographic distribution of similar gonopod morphology. However, the four sequenced “Dargo” males are all phylogenetically closer to *P.jeekeli* in the combined COI and 16S dataset and form a well-supported group (Fig. 3; bootstrap value 98% and with uncorrected percent difference of 2.4%), and appear in the tree next to a population of *P.jeekeli* from northeastern Tasmania. The Dargo collection site is close to the observed species distribution boundary between *P.jeekeli* and *P.nigrovirgatum* (Fig. 4).

### Morphology and sequence analysis of *Somethuscastaneus* from Mount Osmond

**Figure 5.**
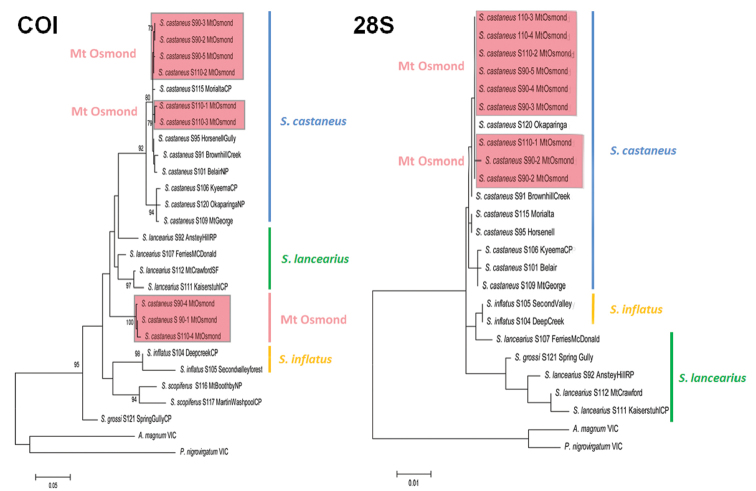
COI and 28S bootstrap consensus trees for *Somethus* species; maximum likelihood, 1000 bootstrap replicates.

All six male specimens from Mt Osmond fully agree in gonopod morphology with *S.castaneus* and lack a medial prefemoral process (Decker 2016b). In the 28S phylogenetic analysis, all nine sequenced specimens (uncorrected p-distance of 0–0.08%) occur within a clade of *S.castaneus* and are closest to specimens from Onkaparinga, Horsnell Gully Conservation Park, and Brownhill Creek Recreation Park (Fig. 5). In the COI analysis (Fig. 5), three specimens from the northern slope of Mt Osmond (site S90) and three from the southwestern slope (site S110) occur with *S.castaneus* from Morialta Conservation Park, Brownhill Creek Recreation Park, Horsnell Gully Conservation Park, and Belair National Park (uncorrected p-distances 0.3–1.1%).

**Figure 6.**
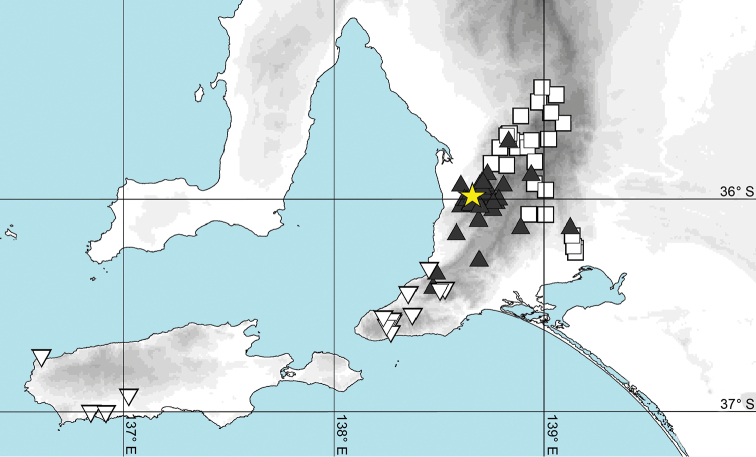
Map of South Australia and southern Mt Lofty Ranges showing localities for *Somethus* species: *S.castaneus* (filled triangle), *S.inflatus* (open triangle), *S.lancearius* (square), and *S.castaneus* from Mt. Osmond (yellow star).

However, two specimens from site S90 and one from site S110 form a separate, well-supported clade (100% bootstrap support) within *Somethus*, with genetic p-distances of 4.8–6.9% to *S.lancearius*, 5.3–6.9% to *S.castaneus* and 7.2–8.8 % to *S.inflatus*. The two Mt Osmond localities are in the centre of the *S.castaneus* distribution (Fig. 6).

## Discussion

### Pogonosternumcf.nigrovirgatum “Dargo”

Despite the clear phylogenetic placement of this form within *P.jeekeli* as indicated by COI+16S gene trees (Fig. 3), the gonopod morphology of the seven Dargo specimens is closest to that of *P.nigrovirgatum*. In addition, tibial and tarsal brushes in the Dargo males extend only to leg pair 7, whereas in *P.jeekeli* the brushes reach leg pair 9 (74 studied males, Decker et al. 2017). In *P.nigrovirgatum* male brushes typically extend to leg pair 7, and infrequently to leg pair 9 in the area around Port Philip Bay.

*Pogonosternumnigrovirgatum* and *P.jeekeli* form a sister clade (Decker 2016a) and the Dargo site is located close to the presumed boundary between the species (Fig. 4). The observed discordance between morphology and genetics could be the result of introgressive hybridisation of *P.jeekeli* and *P.nigrovirgatum*, resulting in a *P.nigrovirgatum*-type gonopod phenotype but a *P.jeekeli* mitochondrial COI and 16S genotypes. Furthermore, the finding of seven discordant males with a *P.nigrovirgatum*-type gonopod shows that the form is not the result of a developmental aberration in an individual within a *P.jeekeli* population. Another possibility is that P.cf.nigrovirgatum “Dargo” is simply a local variant of *P.jeekeli*, a species which is known to have variable gonopods in a genus that exhibits high gonopod variability (Decker 2016a, Decker et al. 2017). However, *P.jeekeli* is the only *Pogonosternum* species with tibial and tarsal brushes always ranging to leg pair 9. In contrast, brushes to leg pair 7 are found in *P.adrianae* Jeekel, 1982, *P.montanum* Decker, 2017, most males of *P.nigrovirgatum* and some males of *P.laetificum* Jeekel, 1982. This leg pair 7 limit is observed in four out of the five species in the genus *Pogonosternum* and likely to be plesiomorphic. Thus the hypothetical local variation in *P.jeekeli* resulting in the “Dargo” phenotype would involve both a change in gonopod morphology and a regression from apomorphy or common distribution of tibial and tarsal brushes in *P.jeekeli*. However, the author favours the hypothesis that two closely related *Pogonosternum* species have undergone introgressive hybridisation in an area of range overlap near Dargo. To distinguish between hypotheses of introgression versus gene tree lineage sorting, further gene sampling and analysis is needed of *Pogonosternum* individuals near the Dargo site and along near the presumed distribution boundary between *P.nigrovirgatum* and *P.jeekeli*. In addition, sampling of *P.jeekeli* and *P.nigrovirgatum* far from sympatry are needed.

### *Somethuscastaneus* from Mount Osmond

*Somethuscastaneus* was sampled for sequencing at 13 localities covering most of the known species range, and genetic variability was shown to be low (up to 3.8% in uncorrected p-distances in COI) with three phylogenetic lineages (Decker 2016b). The morphologically typical *S.castaneus* from Mt Osmond, in the centre of the species range, includes three individuals whose COI sequences are not close to those of either the other *S.castaneus*, *S.inflatus* or *S.lancearius*, although they are slightly closer to those of *S.lancearius* (in % bp difference). *Somethuslancearius* is distributed in the north-eastern and eastern part of the Adelaide Hills, with some scattered, possibly introduced, occurrences of *S.castaneus* within its distributional area. The closest record of *S.lancearius* is about 17 km from Mt Osmond. Genetic variability within *S.lancearius* from five sampled localities is 1.6–5.8% (in uncorrected p-distances in COI) and with unique haplotypes that correspond to geographical areas (Decker 2016b). Widespread sampling of this species is no longer possible, as natural vegetation has largely been cleared within its range and *S.lancearius* is now restricted to scattered conservation areas and tiny remnants.

Several of the paratypes of *S.inflatus* were collected in the Adelaide suburb of Glen Osmond, near Mt Osmond, in 1969 (Jeekel 2002). These paratypes appear to have been lost and could not be compared with recently collected *S.inflatus* (Decker 2016b), and no *S.inflatus* have since been found on Mt Osmond. *S.inflatus* is distributed to the southwest of Adelaide on the Fleurieu Peninsula and on Kangaroo Island (Fig. 6). Jeekel’s *S.inflatus* paratypes might represent a non-permanent introduction.

The three discordant *S.castaneus* found on Mt Osmond might be evidence for past introgression of mitochondrial DNA following hybridisation with another South Australian *Somethus* species. Alternatively, the anomalous individuals might represent a distinct and distantly related *S.castaneus* lineage which is either naturally occurring on Mt Osmond or introduced from another locality within the Adelaide Hills. There is no support for both hypotheses, but it seems that the likelihood of the presence (or former existence) on Mt Osmond of the in COI and 28S variable *S.lancearius* or *S.inflatus* is higher than that of a fourth distinct COI and 28S lineage in *S.castaneus*.

Future investigations with additional molecular markers and more individuals of from *S.lancearius* may not assist in clarifying the situation, as much of the former genetic variation of *S.lancearius* has probably been lost due to habitat loss and local extinctions. If the missing paratypes of *S.inflatus* are found in future, it might be possible to extract DNA and obtain sequences from them which could reveal whether *S.inflatus* in the Mt Osmond area has contributed mitochondrial COI to the local *S.castaneus* population.

## Conclusion

The results presented here suggest that introgressive hybridisation may have occurred in the paradoxosomatid millipede genera *Pogonosternum* and *Somethus* in southeastern Australia. With the increasing use of molecular data in taxonomy and in barcoding projects, similar cases are likely to be found elsewhere. Interestingly, no evidence of introgressive hybridisation was found in more than 2000 COI sequences from Central European millipedes during the German Barcoding of Life Project (GBOL) (Wesener, Spelda, Reip, Decker pers. comm.). The phenomenon may be rare, or limited to narrow parapatric zones, as appears to be the case in *Pogonosternum*.
